# Antitumoral Activity of Snake Venom Proteins: New Trends in Cancer Therapy

**DOI:** 10.1155/2014/203639

**Published:** 2014-02-13

**Authors:** Leonardo A. Calderon, Juliana C. Sobrinho, Kayena D. Zaqueo, Andrea A. de Moura, Amy N. Grabner, Maurício V. Mazzi, Silvana Marcussi, Auro Nomizo, Carla F. C. Fernandes, Juliana P. Zuliani, Bruna M. A. Carvalho, Saulo L. da Silva, Rodrigo G. Stábeli, Andreimar M. Soares

**Affiliations:** ^1^Centro de Estudos de Biomoléculas Aplicadas à Saúde, CEBio, Fundação Oswaldo Cruz, Fiocruz Rondônia e Departamento de Medicina, Universidade Federal de Rondônia, UNIR, Porto Velho, RO, Brazil; ^2^Fundação Hermínio Ometto, UNIARARAS, Núcleo de Ciências da Saúde-NUCISA, 13607-339 Araras, SP, Brazil; ^3^Departamento de Química, Universidade Federal de Lavras, UFLA, 37200-000 Lavras, MG, Brazil; ^4^Departamento de Análises Clínicas, Toxicológicas e Bromatológicas, Faculdade de Ciências Farmacêuticas de Ribeirão Preto, Universidade de São Paulo, USP, Ribeirão Preto, SP, Brazil; ^5^Departamento de Química, Biotecnologia e Engenharia de Bioprocessos, Universidade Federal de São João del Rei, UFSJ, Campus Alto paraopeba, Ouro Branco, MG, Brazil

## Abstract

For more than half a century, cytotoxic agents have been investigated as a possible treatment for cancer. Research on animal venoms has revealed their high toxicity on tissues and cell cultures, both normal and tumoral. Snake venoms show the highest cytotoxic potential, since ophidian accidents cause a large amount of tissue damage, suggesting a promising utilization of these venoms or their components as antitumoral agents. Over the last few years, we have studied the effects of snake venoms and their isolated enzymes on tumor cell cultures. Some *in vivo* assays showed antineoplastic activity against induced tumors in mice. In human beings, both the crude venom and isolated enzymes revealed antitumor activities in preliminary assays, with measurable clinical responses in the advanced treatment phase. These enzymes include metalloproteases (MP), disintegrins, L-amino acid oxidases (LAAOs), C-type lectins, and phospholipases A_2_ (PLA_2_s). Their mechanisms of action include direct toxic action (PLA_2_s), free radical generation (LAAOs), apoptosis induction (PLA_2_s, MP, and LAAOs), and antiangiogenesis (disintegrins and lectins). Higher cytotoxic and cytostatic activities upon tumor cells than normal cells suggest the possibility for clinical applications. Further studies should be conducted to ensure the efficacy and safety of different snake venom compounds for cancer drug development.

## 1. Introduction

Cancer is a chronic degenerative disease considered to be the second most common cause of death in economically developing countries [1, 2]. According to a recent report by the International Agency for Research on Cancer (IARC), there are currently more than 10 million cases of cancer per year worldwide. In 2008 alone there were 12.7 million new cases of cancer worldwide and the WHO estimates that the disease will cause about 13.1 million deaths by 2030 [3].

Cancer is characterized by an accelerated and uncontrolled multiplication of a set of aberrant cells which lose their apoptotic ability. Research has been undertaken in order to find out the factors which promote uncontrolled multiplication of cells and how cancer genes affect cell signaling, chromatin, and epigenomic regulation and RNA splicing, protein homeostasis, metabolism, and lineage maturation [4–6].

Understanding the events that transform a normal cell into a cancer cell has caused new therapies to develop that are more precisely designed to treat a critical gene or biological pathway [7]. Based on their mechanism of action, antitumor drugs that target the cell cycle can be divided generally into three categories, namely, blocking DNA synthesis, causing DNA damage, and stopping mitosis [8]. However, cancer therapy continues involving invasive procedures, including catheter application of chemotherapy, surgery to remove the tumor(s), the use of radiation, and even nonselective cytotoxic drugs [9, 10]. Therefore, the search for new active drugs for cancer therapy is one of the goals of biotechnological research. The expansion of new drugs in oncology represents one of the most promising objectives of the pharmaceutical industry. Many of these compounds are derived from the extraction and purification of toxins and secondary metabolites originating from microorganisms, plants, and animals [11, 12].

Several compounds from venomous animals, such as snakes, spiders, scorpions, caterpillars, bees, insects, wasps, centipedes, ants, toads, and frogs, have largely shown biotechnological or pharmacological applications [13–17]. Numerous examples may be mentioned. Compound TM-601, a modified form of the peptide Chlorotoxin (CTX), isolated from *Leiurus quinquestriatus* scorpion venom, has been shown to bind specifically to glioma cell surfaces as a specific chloride channel blocker and is currently in phase II of human trials [18, 19]. Another example is the venom-derived drug Prialt (ziconotide) generated from the venom peptide of the marine snail *Conus magus* [20] and the drug Byetta (exenatide), a synthetic version of exendin-4 utilized in the treatment of Type 2 diabetes, from the saliva of the Gila monster lizard [21, 22].

The ability of some snake venom toxins to cause toxicity is associated with their high specificity and affinity for cell and tissues. In spite of their toxicological effects, several isolated snake venom proteins and peptides have practical applications as pharmaceutical agents [23]. For example, thrombolytic agents have been used in several cases of vascular disorder [24], antimicrobial activity against gram-positive and gram-negative bacteria [25, 26], antiviral activity against several types of viruses including the herpes simplex virus [27], yellow fever and dengue [28], antiparasitic activity against *Leishmania* [29] and *Plasmodium falciparum* [30], and antifungal activity [31], among other examples.

For cancer treatment, there is great interest in drug design, providing structural templates for the study of new molecules or cellular mechanisms. The use of snake venom in the treatment of some diseases began about sixty years ago in folk medicine. Thus, the biological and toxicological mechanisms involved in snakebites led physicians to study new methods on the isolation of venom constituents, as well as to understand how these compounds could help in medicine.

## 2. Antitumoral Activity of Snake Venoms

**Figure 1:**
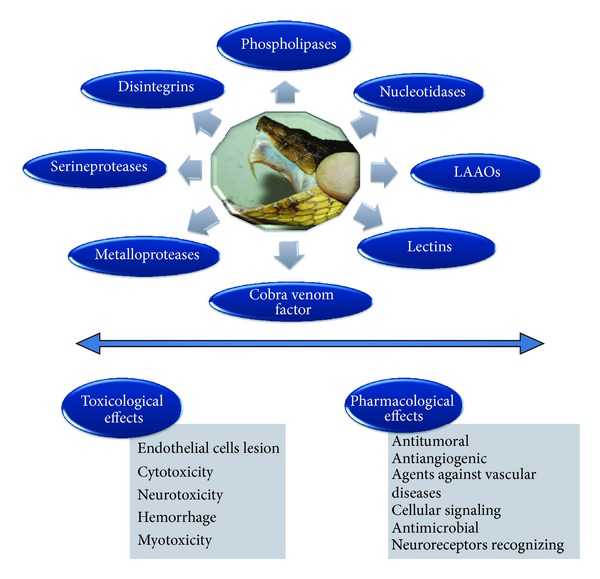
The wide spectrum of action and recent applications of snake venom toxins. The figure depicts the paradigms between toxicological and pharmacological effects of isolated toxins. Different cellular targets are related to different kinds of mechanisms.

Snake venom is a complex mixture of different components that include peptides, proteins, enzymes, carbohydrates, and minerals. Inside a group of enzymes may be found acetylcholinesterases, L-amino acid oxidases, serineproteases, metalloproteases, and phospholipases A_2_ [32] (Figure 1). The cytotoxicity of snake venoms is related to cellular metabolism alterations with a major effect on tumor cells when compared with normal cells. This observation stimulated the development of most chemotherapeutic drugs based on toxins produced in animals, which have the capacity to be highly cytotoxic.

The ability of snake venoms to act upon tumor cells has been known for a long time. The first reported studies on using snake venom against tumor cells were related to the defibrination process. It was suggested that Ancrod, a polypeptide from *Agkistrodon rhodostoma*, administered with cyclophosphamide, could produce defibrination, thus decreasing the tumor weight by fibrinolysis and contributing to both detachment and decreased spread of some tumors [33]. However, their results showed that, besides defibrination, other complex mechanisms including platelet aggregation could be involved in the process.

Braganca et al. [34, 35] assayed a small fraction of *Naja naja* venom on cell cultures of Yoshida sarcoma, calling it cobra venom factor (CVF). Kaneda et al. [36] studied the antitumoral potential of purified peptides (cardiotoxin and cytotoxin) from the *Naja naja atra* snake. Then, Chiam-Matyas and Ovadia [37] showed the cytotoxic properties of several crude venoms from terrestrial snakes with lytic effects on cultures of malignant melanoma tumor cells.

In the past, snake venoms were used to understand the molecular mechanism of some receptors, such as acetylcholine (ACh), and their involvement with some diseases. Two groups of toxins (*α*-BuTX and Erabutoxin and b—ETXa and ETXb) isolated from *Bungarus multicinctus* and *Laticauda semifasciata*, respectively, showed high affinity toward normal and tumor cells, displaying both cytolytic and cytotoxic effects. Interactions of such toxins with ACh led to the application of these compounds as probes not only to elucidate neurophysiology but also to study some tumor cells. Although *α*-BuTX inhibited neuroblastomas, it was too toxic for *in vivo* assays [38]. Moreover, no relationship was observed between a cytotoxic effect and ACh receptors [39].

Experiments have shown that cytotoxic effects displayed by snake venoms are specifically related to the species, genus, and tissue targets. Thus, snake venoms were grouped according to their pathophysiological activities as follows: (i) venoms which cause irreversible alterations on the cell, totally destroying it (this group includes Elapidae venoms); (ii) Crotalidae venoms which cause loss of the cell process viability; and (iii) Viperidae venoms, which cause alterations of cell aggregation. *In vivo*, it was demonstrated that the venom of *Naja nigricollis* inhibited the growth of melanoma through one of these mechanisms. Thus, these findings gave new direction and probable application of snake venom as well as isolated toxins for cancer treatment [40].

Snake venom toxins were also investigated as blockers of metastasis. Metastasis is one of the major causes of death in patients with cancer, being dependent on steps such as adhesion, migration, invasion of blood or lymph vessels, exiting the vessel (with the help of matrix metalloproteinases—MMPs), and finally interaction with the tissue target [41]. Yang et al. [42] studied an inhibitor of integrins that is an important class of cell surface receptors, critically involved in cell-cell and cell-matrix interactions. Particularly, the subfamilies *β*1 and *β*3 play a key role in tumor invasion and dissemination. The group isolated contortrostatin, a disintegrin from *Agkistrodon contortrix contortrix* venom, which is a potent inhibitor of *β*1-integrin-mediated adhesion in human metastatic melanoma cells. Cardiotoxin III (CTX-III) isolated from *Naja naja atra* in the study by Jokhio and Ansari [43] also demonstrated antimetastatic potential by decreasing the expression and activity of matrix metalloproteinase MMP-9, caused by the inactivation of p38 MAPK and PI3K/Akt signaling pathways and NF-*κ*B activity. This suppressive effect assists in inhibiting the migration and invasion of cells causing breast cancer.

As of the last decade, a new strategy has been applied to research on snake venoms with antitumor action, with the focus not only on identifying components with this feature but also on understanding the mechanism of action of toxins that reduce cancer. Several mechanisms of action have been related, as in the study of a cardiotoxin that induces apoptosis in K562 cells through an ROS-independent mitochondrial dysfunction pathway and the caspase-dependent mechanism of Bax/Bcl-2 ratio in human colorectal Colo205 cancer [44]. Juhl et al. [45] described the feasibility of using snake venom in suppressing breast cancer tissue through the inhibition of nucleic acid synthesis. This study shows that snake venom strongly inhibited the formation of nucleic acids in breast cancer tissues. It may cause a decrease in cell proliferation.

The ability of snake venom toxins to destroy malignant cells or to share cytotoxic activity was interesting in areas such as immunology. The use of monoclonal antibodies as antitumor therapeutic agents has not been very promising. However, in coupling a nontoxic CVF isolated from *Naja naja siamensis* to monoclonal antitumor antibodies, several nontoxic antibodies were activated and converted into cytotoxic compounds [46, 47]. Thus, these antibody-CVF conjugates might be a promising therapeutic approach, mediating selective complement-dependent agents of human melanoma, leukemia, and neuroblastomas. Later, it was confirmed that CVF is an important factor for the synthesis of immunoconjugates, which are more specific towards carcinoma cells [48]. In another study using cytotoxin P4, isolated from the same snake, primary conclusions showed for the first time, *in vitro* and *in vivo*, that this peptide caused histopathological changes in leukemia cells and specifically in organelles such as mitochondria [47, 48]. Cytotoxins CT1 and CT2 from *Naja oxiana*, CT3 from *Naja kaouthia, *and CT1 from *Naja haje *were demonstrated to possess this property against human lung adenocarcinoma A549 and promyelocytic leukemia HL60 cells [49].

There are studies showing that *Bothrops jararaca* venom (BjV) induces inhibition of Ehrlich ascites tumor (EAT) growth, accompanied by an increase of mononuclear (MN) leukocytes in all groups inoculated with EAT and/or venom [50]. Different effects were reported with *Crotalus durissus terrificus* venom, one of which was analgesic activity. Zhang et al. [51] showed that the administration of crotoxin to cancer patients reduced the consumption of analgesics.

**Figure 2:**
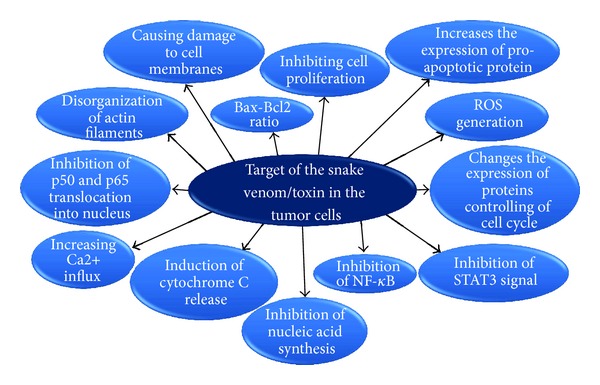
Actions triggered by venoms and/or snake toxins which cause an antitumor effect.

Several studies suggest the application of snake venom toxins for the treatment of animal tumors. Despite several findings and much evidence, there is much controversy regarding this subject. New advances in cellular and molecular biology, as well as biotechnology, focus on the need to understand new mechanisms displayed by snake venom toxins (Figure 2).

## 3. “Targets” in Tumor Cells

Understanding snake venom toxins not only helps relieve the healthcare burden of snakebites but also contributes significantly to the treatment of many other medical conditions. In the early 20th century, the idea of utilizing purified toxins as a source of therapeutics emerged [21]. Anticancer drug developments from natural biological resources are ventured throughout the world. The biodiversity of venoms or toxins makes them a tool from which new therapeutic agents may be developed. Snake venom has been shown to possess a wide spectrum of biological activities. Anticarcinogenic activities of snake crude venoms have been recognized, and their components, including cytotoxins, have been isolated and characterized. These components exhibit various physiological effects such as cytotoxicity, inhibition of platelet aggregation, cardiac arrest, and hemolysis [20].

One of the targets investigated is integrins. They are cell surface adhesion molecules coupling the extracellular environment to the cytoskeleton and are also receptors for transmitting important signals for cell migration, invasion, proliferation, and survival. At least six integrin inhibitors are being evaluated in clinical trials for cancer. The parallel development of integrin antagonists as imaging tools for patient selection may accelerate the discovery of new ways for their use [52].

Integrins play multiple important roles in cancer pathology including tumor cell proliferation, angiogenesis, invasion, and metastasis. The inhibition of angiogenesis is one of the most heavily explored treatment options for cancer, and snake venom disintegrins represent a library of molecules with different structures, potencies, and specificities and are good starting points for developing antiangiogenesis therapeutics [21, 53–55].

Recently, Bazaa et al. [56] characterized MVL-PLA_2_, a novel phospholipase A_2_ from *Macrovipera lebetina* venom, reporting that it exhibited anti-integrin activity. Chwetzoff [57] studied the cytotoxic activity of a basic phospholipase A_2_ from *Naja nigricollis* venom on different cell types and its esterase activity. The cytotoxicity observed was not due to a contaminant, since that would have been eliminated after immunoprecipitation of the basic phospholipase A_2_ by specific monoclonal antibodies. All eukaryotic cells tested were sensitive to the cytotoxic action of the basic phospholipase A_2_. In contrast, the *Escherichia coli* K-12 wild strain was resistant. Thus, the participation of cell membranes in whether the cell is sensitive or resistant to phospholipase A_2_'s attack was investigated using *E. coli* K-12 membrane mutants, and some of them were sensitive. Whether or not esterase activity was required for phospholipase A_2_'s cytotoxic attack was dependent on the cell line tested. Indeed, when the esterase activity of the basic PLA_2_ was eliminated by treatment with p-bromophenacyl bromide, the enzyme retained cytotoxic potency inducing necrosis of certain tumor cells grown *in vitro*, but not of other cells, such as erythrocytes, for which concomitant esterase activity was also necessary. *In vivo* toxicity studies showed that the loss of either cytotoxic potency or esterase activity eliminated the lethal character of the basic PLA_2_. This leads to the proposal that the *in vivo* phospholipase A_2_ toxicity depends on the simultaneous expression of esterase activity and a nonenzymatic property, manifested by the *in vitro* cytotoxic potency.

da Silva et al. [58] demonstrated that *Bothrops jararaca* venom (BjV) has an antitumoral effect on Ehrlich ascites tumor (EAT) cells and induces an increase of polymorphonuclear leukocytes in early stages of tumor growth. The study reported that this venom presents an important inflammatory effect when inoculated in animal models and in human snakebites, and that cytokine levels have been detected in these cases. To evaluate whether the cytokines are involved with the suppression of the tumor's growth, the authors evaluated the cytokine profile in the peritoneal cavity of mice inoculated with EAT cells and treated with BjV. It was observed that EAT implantation induces IL-6, IL-10, and tumor necrosis factor-alpha (TNF-*α*) production and that the treatment with BjV suppresses production of these cytokines. Furthermore, it was suggested that the IL-6 detected in the present study was produced by the EAT cells and the suppression of its production could be associated with the antitumoral effect of BjV.

Pituitary adenomas are neuroendocrine tumors that produce different endocrine and metabolic alterations, including hyperprolactinemia, acromegaly, and Cushing's disease. These different clinical features of pituitary tumors are the result of the overproduction of hormones by the different pituitary cell types. Recent advances in the understanding of the signaling pathways that control hormone production in pituitary cells provide a source of potential therapeutic targets. Therefore, the study of signaling pathways that control hormone production and proliferation is a good source of candidate targets in pituitary tumors [59].

Structural and functional investigations of these proteins and enzymes from snake venoms may contribute to the advancement of toxinology and to the elaboration of novel therapeutic agents [60].

## 4. Snake Venom L-Amino Acid Oxidases (svLAAOs)

L-amino acid oxidases (LAAO) are enzymes diffusely distributed in several organisms, such as bacteria, fungi, algae, and snakes. They are dimeric flavoenzymes, which catalyse the oxidative deamination of L-amino acids into ammonia, alpha-keto acids, and H_2_O_2_ through an intermediary amino acid. These glycoproteins are typically found in the homodimeric form accompanied by a cofactor, which can be flavin adenine dinucleotide or flavin mononucleotide. LAAOs are also found in venoms of several snake species [61]. They are purified generally in either acidic or basic form, with an isoelectric point between 4.4 and 8.5, having FMN and FAD as cofactors (approximately 2 mol/mol), with a relative molecular mass of 120.000–150,000 in native form and 55,000–66,000 in its monomeric form [61–68].

Until the nineties, researchers were restricted to the study of structural and functional characteristics of these enzymes [61]. From this decade, the correlation between the production of L-amino acid oxidases and their use in the metabolic pathways that involve nitrogen, as well as the production of hydrogen peroxide and ammonia, started to represent a horizon in the development of new biotechnological applications.

The high toxicity presented by this class of enzymes is not yet completely understood. Hypotheses have been studied through interaction with cell membrane receptors, which have the potential to produce high hydrogen peroxide concentrations [69]. The first function of LAAO is probably to promote hypotension in the victim by activating soluble guanylate cyclase in the presence of superoxide dismutase [70]. It has been demonstrated that the isolated enzymes of *C. adamanteus* and *C. atrox* can interact specifically with mammal endothelial cells possibly by increasing hydrogen peroxide production [61, 71]. Snake venom LAAOs and their studies in structural and molecular biology have been very important for pharmacology [61, 72]. They have been characterized through their different functions, such as substrate preference, apoptosis induction, cytotoxicity, hemolysis, activation or inhibition of platelet aggregation, hemorrhage induction, edema, and bactericidal activity [61, 68, 73, 74]. Hemorrhage is a common phenomenon caused by snake venom LAAOs, which unchains complex processes, such as apoptosis of endothelial or vascular cells [61].

Many research groups describe LAAOs as apoptosis inducers, in human embryonic cells (293T) [75], human promyelocytic leukemia cells (HL-60) [65, 76, 77], human monocytic cells (MM6) [68], rat lymphocytic leukemia cells (L1210), and human leukemia T cells [71].

In this review, we will focus on the results obtained through several assays regarding their cytotoxic effect upon cell cultures and animal models, as well as the mechanisms involved and reactions able to explain these effects.

Araki et al. [78] showed that cytotoxic substances in the venoms of *C. atrox*, *T. flavoviridis*, *G. h. blomhoffii*, *V. ammodytes,* and *B. arietans* cause apoptosis in cell lines in a selective way: being active on vascular endothelial cells of the human lung and inactive on the same cell line of rats, smooth muscle cells of bovines, and embryonic fibroblasts of human beings [70].

Interestingly, the assays that evaluated the cytotoxic activity of LAAOs attributed the apoptotic effect to H_2_O_2_ produced by the oxidative reaction, but other studies showed that different mechanisms might exist [71]. Several mice tumor cell lines were assayed for LAAO from *Agkistrodon halys* with high apoptosis induction, even at low concentrations. In the presence of the enzyme, cultured L1210 cell nuclei were split and showed the characteristic ladder-like pattern of DNA fragmentation. The enzyme binds directly to the cell surface, thereby increasing the local concentration of H_2_O_2_. However, experimental evidence suggests that the apoptotic mechanism induced by LAAO is distinguished from the one caused by exogenous H_2_O_2_ [71].

In 1997, the Korean group of Ahn et al. [79] published an interesting study investigating the LAAO from *Ophiophagus hannah*, starting from its purification, biochemical characterization up to its cytotoxic activity upon several tumor cell cultures, both human and murine, achieving around 74% of inhibition of tumor proliferation at a concentration of 2 *μ*g/mL. A different mechanism for H_2_O_2_ production was also postulated, by inhibition of thymidine incorporation, with a consequent interaction with DNA. Markland [80] suggested that this enzyme probably prevents the adhesion of tumor cells and the formation of metastasis in the host by inhibition of platelet aggregation and activation of phagocytic cells from the immunological system.

In 1999, Souza et al. [67] showed the cytotoxicity level of an LAAO from *Agkistrodon contortrix laticinctus* through the fragmentation of DNA on HL-60 cultures hybrid cells. Results showed signs of induction of apoptosis after extraction of DNA. Apoptosis related to svLAAO activity is a polemic subject since some authors postulate that this activity derives from H_2_O_2_ action from the enzymatic reaction. Suhr and Kim [71] already showed that apoptosis induction was not related to H_2_O_2_ alone. Dipietrantonio et al. [81] detected an increase of caspase 3 activity in HL-60 cells exposed to H_2_O_2_. Caspases are proteases of the cysteine family that are commonly apoptosis markers.

Suhara et al. [82] found that H_2_O_2_ induces the regulation of the TNF receptor superfamily (FAS) in human endothelial cells and that the activation of the protein tyrosine kinase may be involved in the expression of FAS induced by H_2_O_2_. Thus, apoptosis mediated by FAS in human endothelial cells can contribute to the mechanism of H_2_O_2_ in inducing cellular damage.

Stábeli et al. [83] showed that inhibition of the toxic effect of LAAO from *Bothrops moojeni* was retained when catalase, an H_2_O_2_ scavenger, was added. This same enzyme showed a cytotoxic effect upon Ehrlich ascite tumor cells and showed efficiency as a bactericidal, trypanocidal, leishmanicidal and apoptotic agent through DNA fragmentation. The same observation was made regarding other isoforms of this enzyme, isolated from the same species, on leishmanicidal activity [84].

BjarLAAO-I, an L-amino acid oxidase from *Bothrops jararaca* snake venom, was purified by de Vieira Santos et al. [85]. This LAAO inhibited Ehrlich ascites tumor growth and induced an influx of polymorphonuclear cells, as well as spontaneous liberation of H_2_O_2_ from peritoneal macrophages. Later, BjarLAAO-I induced mononuclear influx and peritoneal macrophage spreading but the mechanisms that inhibit tumor growth have not been clarified. Animals treated with BjarLAAO-I showed higher survival time.

Zhang et al. [76] evaluated the activity of an LAAO from *Trimeresurus stejnegeri* as an antiviral agent, as well as the cytotoxic effect of this enzyme upon lymphocytic leukemia C8166 cells, discussing the role of H_2_O_2_ in cytotoxic activity. Using catalase, an H_2_O_2_ scavenger, these authors observed that LAAO greatly lost its activity, but even in the absence of H_2_O_2_ cytotoxicity was still significant, supporting the hypothesis that other mechanisms of action are probably involved. This was already postulated by the assay that evaluated the bactericidal activity of the mouse milk enzyme as a protecting agent of mastitis [86].

In investigating apoxin, an LAAO from *Crotalus atrox*, Torii et al. [75] concluded that H_2_O_2_ indeed played an important role in apoptosis induction. Thus Ali et al. [68] showed that the studies with LAAO from *Eristicophis macmahoni* reinforce the already proposed theory of the participation of H_2_O_2_, produced during the enzyme's activity, in biological and pharmacological effects, such as apoptosis, cytotoxicity, bactericidal activity, and platelet aggregation induction.

Sun et al. [87] showed the antitumoral effect on an LAAO from *Trimeresurus flavoviridis* at several concentrations upon human glioma cell cultures. They evaluated apoptosis using flow cytometry, showing the inhibitory effect of the enzyme in the presence of catalase. Once more it was shown that concentrations as low as 10 *μ*g/mL were able to destroy 90% of the cells. However, in addition to inhibition by catalase, there was still apoptosis, probably related to the binding of the enzyme to the cell membrane.

Bp-LAAO, an L-amino acid oxidase from *Bothrops pauloensis* snake venom purified by Rodrigues et al. [60], showed dose-dependent leishmanicidal, bactericidal, and antitumoral activities. This antitumor activity was observed in human breast cancer cells (SKBR-3), acute T leukemia cell (JURKAT), and Ehrlich ascitic tumor (EAT) cell lines. Moreover Bp-LAAO induced platelet aggregation in platelet-rich plasma by inhibiting catalase.

In 2012, LAAOs isolated from *Ophiophagus hannah *venom decreased thymidine uptake in murine melanoma, fibrosarcoma, colorectal cancer, and Chinese hamster ovary cell line and also showed reduction in cellular proliferation [79]. In addition, an LAAO isolated from *Agkistrodon acutus *snake venom showed an accumulation of tumor cells at the sub-G1 phase of the cell cycle. It also induced apoptosis via the Fas pathway in A549 cells (human alveolar epithelial cell line) [88–90].

## 5. Snake Venom Phospholipases A_**2**_ (svPLA_**2**_s)

Phospholipases A_2_ (PLA_2_s) are enzymes of high medical-scientific interest due to their involvement in several inflammatory human diseases and in envenomation by snake and bee venoms. PLA_2_s also play an important role in diet lipid catabolism and in the general metabolism of lipid membranes. In addition, arachidonic acid, one of their hydrolysis products, is the precursor of important eicosanoids displaying prominent biological activities, namely, prostaglandins, prostacyclins, thromboxanes, and leucotrienes. PLA_2_s constitute a super-family of different enzymes belonging to four groups based on their source, amino acid sequences, and biochemical characteristics [91–93].

Altered lipid biosynthesis and deregulated lipogenesis are typical features of cancer. Consequently, these pathways have been investigated as novel therapeutic targets. Lipolytic phospholipase A_2_ (PLA_2_) enzymes have been explored as novel anticancer agents [94–96].

Different types of phospholipases have been shown to possess antitumor and antiangiogenic properties, such as acidic and basic PLA_2_s, and synthetic peptides derived from PLA_2_ homologues [97–100]. Recently, two phospholipases A_2_ from *Cerastes cerastes *venom, CC-PLA_2_-1 and CC-PLA_2_-2, were purified and characterized. They were able to inhibit cancerous cell adhesion and migration, along with angiogenesis, both *in vitro* and *in vivo* [101, 102]. Phospholipase A_2_ from *Macrovipera lebetina transmediterranea* venom (MVL-PLA_2_) inhibited tumor cell adhesion and migration, as well as angiogenesis. This process occurs through an increase in microtubule dynamics and disorganization of focal adhesions [56, 103].

Some PLA_2_s isolated from Viperidae venoms are capable of inducing antitumoral activity, suggesting that these molecules may be a new class of anticancer agents and provide new molecular and biological insights into cancer drug development [60, 102].

PLA_2_ activity is related to the metabolism of cell membranes. In 1989, Chwetzoff et al. reported that a *Naja nigricollis* PLA_2_, called nigexin, displays important cytotoxicity upon cell cultures of several tumors, such as epithelial, neuroblastoma, and leukemia tumors [104]. Most PLA_2_s do not show this profile and the authors suggest that the enzymatic activity is not responsible for the cytotoxic effect and other mechanisms must be involved.

VRCTC-310-Onco is a pharmaceutical product under development, composed of crotoxin (from *Crotalus durissus terrificus*) and cardiotoxin (from *Naja naja atra*) at an equimolar ratio. Crotoxin B is the main component, a 14 kDa neurotoxic secretory phospholipase A_2_, that, in addition to its classic enzymatic activity, binds and activates cell receptors located in the plasma membrane [105]. By these or other mechanisms, crotoxin interferes with the signaling of the epidermal growth factor receptor [106]. Addition of cardiotoxin dissociates cytotoxicity (required for antitumoral activity) and neurotoxicity (otherwise, its main side effect) and allows a useful concentration to be achieved *in vivo* [107]. Injection of crotoxin into mice has been reported to increase the *in vivo* production of tumor necrosis factor *α* (TNF- *α*) together with the stimulation of the hypothalamic-pituitary axis [108].

Preliminary data showed that a protein fraction of the venom could be a useful tool for cancer therapy. Costa et al. [108] evaluated the probable mechanism of action of this union and concluded that PLA_2_s act on the receptors of the epidermal growth factor. Another route of action might be a decreased production of tumor necrosis factor. In 2002, the same group suggested intravenous (i.v) administration of the drug, and that route did not show toxicity for the kidneys, heart, or lungs [109].

Roberto et al. [97] showed that the *Bothrops jararacussu* acidic PLA_2_, BthA-I-PLA_2_, displays antitumoral effects upon Ehrlich ascites tumor, leukemia (Jurkat), and breast cell lines, using several enzyme concentrations. At 100 *μ*g/mL, the toxicity was close to that of the control drug (methotrexate). This activity seems to be related to apoptosis. It is postulated that PLA_2_s probably speed up the turnover of phospholipids, what may generate typical changes to apoptosis.

Gebrim et al. [110] evaluated both *in vitro* and *in vivo* antitumor activity of BPB-modified BthTX-I (PLA_2_ Lys49) and its cationic synthetic peptide derived from the 115–129 C-terminal region. BPB-BthTX-I presented 70 and 90% cytotoxicity upon Jurkat, B16F10, and S180 tumor cell lines, which were also susceptible to the lytic action of the synthetic peptide. BPB-BthTX-I showed dose-dependent cytotoxicity and this effect was shown to be inferior to that of the native toxin on all tumor cell lines and macrophages.

Several articles state the affinity of secretory PLA_2_s for different membrane receptors and lipids, but since those related to *Bothrops* myotoxins are still unknown, two mechanisms have been proposed to explain Lys49 myotoxin cytotoxicity: a fatty acid-dependent lysis by means of an interaction with a receptor able to activate the myotoxin and the activation of intracellular lipase unleashed by the binding of the myotoxin to the receptor [110–112].

A phospholipase B purified from *Pseudechis colletti* was assayed on rabdomiosarcoma A673 tumor cells. A cytotoxic effect on sarcoma cells was observed, but no lytic activity against fibroblasts was found. This effect was related to destructive action of the enzyme upon the striated muscles [113]. Another study with recombinant sea snake PLA_2_ (rSSBPLA_2_) from *Lapemis hardwickii* venom showed its *in vivo* and *in vitro* enzymatic activity on different tumor cell lines [114].

The synthetic peptides p-AppK and pEM-2 derived from Lys49 phospholipase A_2_ homologues from *Agkistrodon piscivorus piscivorus* snake venom were evaluated against different tumor cell lines (B16 melanoma, EMT6 mammary carcinoma, S-180 sarcoma, P3X myeloma, and tEnd endothelial cells) and showed a rapid cytotoxic effect. In general, peptide p-AppK was slightly more potent than pEM-2 against various tumor cell lines, except for the P3X myeloma cells, which were slightly more susceptible to pEM-2 [98].

Ferguson et al. [96] have proposed dextrin-PLA_2_ as a bioresponsive anticancer therapeutic polymer and a new example of polymer-enzyme liposome therapy (PELT). Cytotoxicity was assessed in MCF-7, B16F10, and HT29 tumor cell lines using an MTT assay. Therefore, prior to designing protocols for *in vivo* studies it was considered important to further investigate the dextrin-PLA_2_ action mechanism, particularly since this could potentially involve multiple cellular targets. Preliminary experiments show that the conjugate is internalized by endocytosis more readily than PLA_2_ alone. The resulting redistribution of intracellular vesicles suggests a multimodal mechanism involving both plasma and intracellular-vesicle membrane interactions.

Documented literature reported that the PLA_2_ from *Macrovipera lebetina* venom exhibits anti-integrin activity. In their study with HMEC-1 (human microvascular endothelial cells), MVL-PLA_2_ has shown inhibition of cell adhesion and migration, as well as disturbed the actin cytoskeleton and the distribution of *α*v*β*3 integrin [56, 90].

Khunsap et al. [100] purified a PLA_2_ (Drs-PLA_2_) from *Daboia russelii siamensis* venom which exhibited indirect hemolytic, anticoagulant, and cytotoxic activities. Moreover, this PLA_2_ decreased human skin melanoma (SK-MEL-28) cell viability in a dose-dependent manner, as well as migration with an IC_50_ of 25.6 nM. Moreover, Drs-PLA_2_ inhibited the colonization of skin melanoma cells (B16F10) in BALB/c mice lungs by 65%.

## 6. Snake Venom Lectins

Lectins, proteins that bind to carbohydrates, are found in several animal and vegetal species. They interfere with tumor cell proliferation and the studies about their relationship with cancer are based on the characteristics of endogenous lectins from tumor cells. Some plant lectins showed an inhibitory effect on human prostatic tumor cells [115].

Lectins are polyvalent carbohydrate-binding proteins of nonimmune origin and have been used extensively as histochemical probes to describe changes in tumor cell surface. These glycoproteins are known to influence the growth of cancer cells. BJcuL, a lectin isolated from *Bothrops jararacussu *snake venom, was purified and functionally characterized, and its effect on the proliferation of a number of established human cancer cell lines was determined. The growth of eight cancer cell lines was inhibited in a dose-related manner in the presence of BJcuL. This lectin was a potent growth inhibitor in renal (Caki-1 and A-498) and pancreatic (CFPAC-1) cancer cell lines, with an inhibitory concentration of 50%. These results suggest that BJcuL lectin is an effective inhibitor of cell growth in some cancer cell lines [116].

In 2001, de Carvalho et al. [117] observed that human metastatic breast cancer (MDA-MB-435) and human ovarian carcinoma (OVCAR-5) cell lines adhere, although weakly, to BJcuL. However, the lectin did not inhibit the adhesion of cells to the extracellular matrix proteins fibronectin, laminin, and type I collagen. Importantly, the viability of these tumor cells, other human tumor cell lines, and bovine brain endothelial cell lines were suppressed by BJcuL. These findings suggest that the lectin BJcuL may serve as an interesting tool for combating tumor progression by inhibiting the growth of tumor and endothelial cells. The integrin family of adhesion receptors play an essential role during tumor progression and thus represent interesting potential targets for the development of new therapeutic agents. More recently, Sarray et al. [118] reinforced the cytotoxicity of BJcuL on tumor cells mainly by altering cell adhesion and inducing apoptosis in gastric carcinoma cells MKN45 and AGS. The authors suggested that BJcuL may compete for binding to the cell surface with extracellular matrix glycoproteins and promote actin disassembly and possibly accelerate cellular detachment from the extracellular matrix. After, it was demonstrated that lebecetin, a C-type lectin isolated from *Macrovipera lebetina* venom, displays anti-integrin activity. Lebecetin inhibited integrin-mediated attachment of various tumor cell lines to different adhesion substrates. This protein was able to inhibit adhesion, migration, and invasion of tumor cells. Apparently, the fact that lectin acts on the integrin domains and its antiproliferative activity was significant. At 10 *μ*g/mL, melanoma and sarcoma cell lines had their proliferative profile strongly inhibited [118].

Sarray et al. (2001) demonstrated that lebecetin also interferes with the adhesion of IGR39 melanoma and HT29D4 adenocarcinoma cells. In fact, these two cancer cell lines tightly adhere to immobilized lebecetin. Lebecetin is also able to strongly reduce IGR39 and HT29D4 cell adhesion to fibrinogen and laminin but not to fibronectin and collagen types I and IV, respectively. Adhesion properties of lebecetin may thus involve integrin receptors [118]. Six years later, the same group [119] presented a second C-type lectin, isolated from the same venom which showed potent inhibition of platelet aggregation and seemingly affected cell adhesion, migration, invasion, and proliferation by inhibiting *α*5*β*1 and *α*v-containing integrins. Moreover, the inhibition of *α*5*β*1 and *α*v integrins is likely due to the binding of venom peptides, as both lebectin and lebecetin coimmunoprecipitate with these integrins. Lebectin and lebecetin were the first examples of venom C-type lectins inhibiting an integrin other than the collagen receptor *α*2*β*1.

In a short communication, Nunes et al. [120] showed the cytotoxic activity on tumor cells and apoptosis in K562 cells of BlL, a galactoside-binding lectin isolated from *Bothrops leucurus* venom. Antitumor activity was verified by phosphatidylserine externalization analysis and mitochondrial membrane potential determination.

Therefore, lectins have been proved to be prospects for potential use in cancer therapy.

## 7. Snake Venom Metalloproteases

Metalloproteases are important compounds of most viperid and crotalid venoms. They can trigger hemorrhage by causing changes in blood coagulation or interaction with the main components of the extracellular matrix such as collagen, laminin, and fibronectin [121–123]. Also known as zinc-proteases, snake venom metalloproteases (SVMPs) are multidomain proteins that, through autoproteolysis, can generate biologically active products [124, 125]. According to their structure, these proteins are classified as either part of the mature P-I class, which has only a metalloprotease domain, P-II, which contains a metalloprotease domain followed by a disintegrin domain, P-III, a metalloprotease, with disintegrin-like and cysteine-rich domains, or P-IV, the heterotrimeric class of SVMPs, which possesses an additional snake C-type lectin-like (snaclec) domain, found close to the carboxyl end of the protease which is now included in the P-III group as a subclass (P-IIId) [125–131].

High molecular weight SVMPs are classified as metalloprotease/disintegrin-like/cysteine-rich (MDC) proteins. The complex hemorrhage mechanism induced by these enzymes has led to the investigation of the relationship between the disintegrin domain and the main components of blood coagulation, especially platelets and integrins *α*, *β* [132]. In addition, the application of these enzymes in platelet physiology research contributed to elucidating other probable mechanisms involved in cellular adhesion, which was widely studied with jararhagin, a high molecular weight hemorrhagic metalloprotease isolated from *Bothrops jararaca* venom [128, 133–136].

Various groups of matrix metalloproteases/ADAMs have been shown to be involved in the formation of new vessels during tumor growth [137]. These multidomain proteins are involved in both cancerous cell proliferation and indeed in cell-cell/cell-ECM adhesion [138–144].

Molecular approaches have been performed with high molecular weight metalloproteases from a number of Viperidae species in order to elucidate the complex integrin-disintegrin interactions. SVMPs containing disintegrin-like domains (PIII/PIIIb class) may play a role in targeting the protein to a particular site in cells such as platelets, and endothelial cells, as well as in integrins, extracellular matrix and other substrates [122, 134, 145–148]. The structural similarity between mammalian MMPs (ADAM) and SVMPs (low and high MMPs), including disintegrins, reinforces the idea that snake venom components captivate medical interest as potential molecules for the treatment of animal tumors [149].

## 8. Disintegrins

Along with metalloproteases, disintegrins are important compounds in most viperid and crotalid venoms. Disintegrins represent a family of nontoxic and nonenzymatic low molecular weight (5–10 kDa) RGD-containing peptides naturally presented in snake venoms or synthetics. Originally, these compounds are characterized by their ability to interact with integrins *α*IIb*β*3, *α*5*β*1, and *α*v*β*3lls expressed by a number of cells including those involved in tumor development and proliferation [150, 151].

Based on binding experiments, *αβ* integrins and their subtypes have been identified as major functional adhesion receptors on tumor cells. Indeed, disintegrins from several snake venoms have revealed new possibilities of uses not only in cardiovascular diseases but also as potent inhibitors of tumor cells [128, 149, 152]. Thus, a number of toxins containing RGD-peptides or RGD-containing disintegrins isolated from snake venoms have also been used to elucidate target receptors in a wide variety of primary cultured tumor cells (Table 1).

Studies with metalloproteases and respective peptides that contain the disintegrin domain called RGD have focused on their antitumoral effects. These peptides can act in the extracellular matrix and play an important role in the evolution of angiogenesis and metastatic dissemination of cancer [152–154].

Venoms of *Trimeresurus* and *Agkistrodon* genera contain peptides that potentially inhibit platelet aggregation [150, 155–161], frequently induced by tumor cells. In order to discover new natural compounds to inhibit tumor cells, Kang et al. [88] showed strong *in vivo* evidence that a disintegrin containing the RGD sequence from *Agkistrodon halys brevicaudus* could suppress tumor angiogenesis through *α*v*β*3 integrin interactions. A number of isolated disintegrins demonstrated potential inhibition of tumor cells: Contortrostatin [162], Eritostatin [163], Rhodostomin [164, 165], Obtustatin [166], Trigramin [167], Salmosin [168], Triflavin [169], Albolabrin [170], and Echistatin [171].

Contortrostatin (CN), a disintegrin containing Arg-Gly-Asp, isolated from *Agkistrodon contortrix contortrix* venom, interacts with different epithelial carcinoma and endothelial cell surface receptors. The anticancer potential of CN, a 13.5 kDa protein, was demonstrated because CN recognizes integrins *α*IIb*β*3; *α*5*β*1; *α*5*β*3; *α*5*β*5. Schmitmeier et al. [172] demonstrated that CN exerted antitumor activity along with tumor necrosis factor (TNF-*α*) on human glioblastoma cell lines. This activity may be related to the fact that CN induced the disruption and prevented the binding of integrins to the extracellular matrix [173]. It has been demonstrated that this peptide inhibits *in vitro* and *in vivo* ovarian cancer dissemination and the recruitment of blood vessels to tumors [162, 174, 175]. This first study reinforces that CN is a potent and important molecule not only for therapeutic use in cancer but also to elucidate the several steps involved in tumor progression and metastasis. In 2004, the pharmacological effectiveness of CN was investigated using a mouse model of human mammary cancer. Clinically, the peptide is nonimmunogenic and stable when submitted to a relevant method of drug delivery, such as the liposomal delivery system. This study also shows the effectiveness of the inhibitory effect of contortrostatin on breast cancer progression in orthotopic and xenographic models, as well as the importance of integrins in cellular migration, invasion, matrix degradation, proliferation, and angiogenesis. This protein binds and affects the function of some integrins expressed in cancer, platelet, and endothelial cell surfaces. Encapsulated liposomes were used to release the active protein at the tumor site, thus having a chemotherapeutic application [176].

Another study using the human metastatic breast cancer cell line MDA-MB-435 in mice revealed that contortrostatin from *Agkistrodon contortrix contortrix* has potent antitumoral and antiangiogenic activities and demonstrated that contortrostatin may have potential as a therapeutic agent for the treatment of malignant gliomas [177].

The exact mechanisms that cause tumor regression in experimental animals after treatment with disintegrins are not well established. However, in addition to cell detachment, antiangiogenic activity is an important characteristic of these groups of peptides. Obtustatin, a disintegrin isolated from the venom of *Vipera lebetina obtusa*, showed significant *in vivo* inhibition of Lewis lung carcinoma growth and interacted selectively with the integrin *α*1*β*1, promoting inhibition of angiogenesis [166]. Rhodostomin, a disintegrin isolated from *Calloselasma rhodostoma* venom, affected tumor formation and the angiogenic process in different ways. It was first demonstrated that rhodostomin caused inhibition of Saos-2 (human osteosarcoma cells), TCIPA (tumor cell-induced platelet aggregation), and adhesion of tumor cells to the ECM, suggesting that host platelets may act as causative agents in tumor formation and metastasis [164, 165]. Yang and Huang [178] also showed that rhodostomin strongly inhibits the adhesion activity of ROS 17/2.8 cells (osteosarcoma cells) by TGF-*β*1 (transforming growth factor-*β*1). In 2001, Huang et al. [179] reported that rhodostomin showed antimetastatic activity due to the fact that it is an antiangiogenic substance, which selectively inhibits basic fibroblast growth factor (bFGF-treated) and the viability of human umbilical vein endothelial cells (HUVEC). The interaction between tumor cells and microvasculature including cell-cell adhesion as well as their migratory activity and angiogenesis was also reported using albolabrin, a disintegrin isolated from *Trimeresurus albolabris* venom. The peptide inhibited the adhesion of melanoma cells to extracellular matrix proteins. Inhibition exerted by albolabrin was dose-dependent and reached 70% for fibronectin and 60% for laminin at 20 *μ*g/mL [170]. At 30 *μ*g/mouse, it inhibits the tumor alone in a dose-dependent manner, showing no damage to animal health. The effect of albolabrin on metastasis was evaluated using a B16-BL6 melanoma line and compared with data from the synthetic RGD peptides. Albolabrin was 2000-fold more effective on the mouse melanoma cells. In addition to its inhibitory effect on platelets, salmosin, a disintegrin isolated from *Agkistrodon halys brevicaudus* venom, acts on tumor growth through an antiangiogenic mechanism [88]. Some studies have reported salmosin activity on melanoma cell metastasis and proliferation. Kang et al. [180] and Chung et al. [181] investigated the action of salmosin on integrin receptors, which are mediators of human tumor cell proliferation. Salmosin significantly inhibited the proliferation of human melanoma cells (SK-Mel-2 and B16 cells) in a dose-dependent manner. These authors also observed that salmosin inhibits the adhesion process via two possible mechanisms: (i) it specifically blocks the adhesion of cells to the ECM, via *α*v*β*3 or *β*1 or (ii) via induction of apoptosis, also blocking the *α*v receptor. A novel disintegrin purified from *Gloydius saxatilis *snake venom, named saxatilin, was able to strongly inhibit tumor growth and may be an alternative procedure for antiangiogenic cancer therapy [182].

In 2009, Colombistatin, a disintegrin that inhibited ADP-induced platelet aggregation, human urinary (T24) and skin melanoma (SK-Mel-28) cancer cell adhesion to fibronectin, and cell migration was isolated from the venom of *Bothrops colombiensis *[183].

Thus, along with metalloproteinases, disintegrins from snake venoms have shown a high potential for treatment against cancer.

## 9. Snake Venom Serineproteases

Serineproteases are enzymes found in microorganisms, plants, and many animals. These enzymes display several biological functions and may be involved with digestion, activation of the complement system, cell differentiation, hemostasis, and others. Serineproteases affect several steps of blood clotting, often not specifically, inactivating blood clotting factors involved with platelet aggregation, clotting, and fibrinolysis [184–186]. The antitumoral potential of these proteins is still not well explored, and there are few studies published on their effects and mechanisms of action.

Markland [153] evaluated the *in vitro* and *in vivo* effects of crotalase, a serine protease from *Crotalus adamanteus*, on the growth of B16 melanoma cells in C57BL/6 mice and concluded that the enzyme was able to inhibit the growth of B16 melanoma cells *in vitro*, did not show cytotoxic or cytostatic effects on cells *in vivo*, and did not significantly increase the survival time of the animals.

The effect of defibrinogenation of batroxobin, a thrombin-like enzyme found in *Bothrops atrox moojeni*, presently *Bothrops moojeni* snake venoms, was studied on artificial lung metastasis in mice. The role of natural killer (NK) cells in the inhibitory effects of defibrinogenation on metastasis was also investigated. The administration of batroxobin had no effect at all on spleen lymphocyte NK activity. These results indicated that defibrinogenation due to batroxobin inhibits lung metastasis, and these effects depend on the NK activity of the host [187].

SVSPs have shown great potential for therapeutic and diagnostic use of coagulant disorders, which has generated a positive outlook regarding their study, even though few studies have been published to date.

## 10. Concluding Remarks 

An uncontrolled rush toward the development of cytotoxic agents, selective for tumor cells, has already started. Several classes of drugs were produced, as happened in the seventies with platinum derivatives able to inhibit the growth of bacterium cultures. In the sixties, we find the first reports of cytotoxic activity of snake venom PLA_2_s on Yoshida sarcoma cell cultures [188]. So far, the association of animal venoms with important tissue damages in ophidian accidents is known and the investigation of active fractions for clinical use started to stand out in the scientific literature.

Nowadays, some works have been published with emphasis on the evaluation of specific points of tumor metabolism, like its immunological aspects and the induction of apoptosis. Cellular proliferation is not the only event to be fought in cancer treatment; the capacity of the tumor to invade adjacent tissues and to create new blood vessels can also be used as targets for new treatments. These relationships between cells and components of the extracellular matrix are fundamental in the events that occur in cancer for the invasion of tumor cells and also for the mechanisms of angiogenesis.

In this review, we focused on the new paradigms of both cytotoxic and pharmacological effects of snake venom toxins. A number of *in vivo* and *in vitro* experiments have demonstrated that snake venoms can contribute to the development of new drugs to combat a number of diseases including cancer. Moreover, from 1980 to the 1990s, snake venom peptides isolated from Viperidae and Crotalidae species and containing RGD/ECD sequences in their structures proved to be invaluable tools to recognize specific structures/receptors of platelets and some kinds of cells, as well as to promote physiological and biochemical changes on a cellular level. To date, these compounds are also complements for new therapeutic strategies in mutagenesis-related diseases.

Disintegrins interact with integrins via glycoprotein receptors located on cellular surfaces, which are related to cell-cell and cell-matrix interactions. These complex mechanisms have led to several studies on the elucidation of events or factors that may affect focal adhesion in cancer. Enthusiastic studies using these promising “Leads” or templates have demonstrated that peptides containing the RGD sequence from several snake venom species have the ability either to inhibit angiogenic activity via subtypes of *αβ* receptors or to exhibit selective apoptotic effects. These observations were essential according to a wide variety of pharmacological targets that make snake venom toxins invaluable sources of binders for studying new drugs and a considerable number of inhibitors [140, 189].

## Figures and Tables

**Table 1:** Antitumor activity of snake venoms and isolated compounds.

	Protein name	Snakes	Cellular target/mechanism	Reference
Phospholipase A_2_ (PLA_2_)	Nigexine	*Naja nigricollis nigricollis *	Cytotoxic, altered cell viability and prevented cell proliferation.	[104]
	BthA-I-PLA_2_	*Bothrops jararacussu *	Effect against breast adenocarcinoma, human leukemia T, and Ehrlich ascitic tumor	[97]
	rSSBPLA_2_	*Lapemis hardwickii* (sea snake)	Antitumor effect	[114]
	PLB	*Pseudechis colletti *	Cytotoxicity	[113]
	PLA_2_	*Agkistrodon piscivorus piscivorus *	Synergistic effects with antineoplastic drugs against S49 lymphoma cells	[98]
	BPB-BthTX-I	*B. jararacussu *	Cytotoxicity on S180 tumor cells	[189]
	CC-PLA2-1 and 2	*Cerastes cerastes *	Antitumor and antiangiogenic activities	[101]
	Drs-PLA_2_	*Daboia russelii siamensis *	Inhibition of SK-MEL-28 cell migration and inhibition of the colonization of B16F10 cells in lungs	[100]
	MVL-PLA2	*Macrovipera lebetina *	Inhibits angiogenesis and induces changes in actin cytoskeleton	[56, 102]

L-Aminoacid oxidases (LAAOs)	LAAO	*Ophiophagus hannah *	Cytotoxicity in stomach cancer, murine melanoma, fibrosarcoma, and colorectal and ovary cell lines	[79]
	LAAO	*Agkistrodon halys *	Apoptosis	[71]
	Apoxin-I	*Crotalus atrox *	Apoptosis	[75]
	LAAO	*Eristicophis macmahoni *	Apoptosis	[68]
	BmooLAAO-I	*Bothrops moojeni *	Cytotoxicity and apoptosis	[83]
	LAAO	*Trimeresurus stejnegeri *	Cytotoxicity	[76]
	LAAO	*Trimeresurus flavoviridis *	Antitumor activity	[87]
	AHP-LAAO	*Agkistrodon halys pallas *	Apoptosis	[190]
	LAAO	*Vipera berus berus *	Apoptosis	[77]
	ACTX-6	*Agkistrodon acutus *	Induces apoptosis in human cervical cancer Hela cell line	[191]
	B1-LAAO	*Bothrops leucurus *	Cytotoxicity in the stomach cancer MKN-45, adenocarcinoma HUTU, colorectal RKO, and human fibroblast LL-24 cell lines.	[192]

Metalloproteases	Crovidisin	*Crotalus viridis *	Detachment of ROS 17/2.8 osteosarcoma cells.	[147]
	Jararhagin	*Bothrops jararaca *	Inhibition of melanoma cells and proapoptotic effect selective, interfering with the adhesion mechanisms	[136]
	leucurolysin-B	*Bothrops leucurus *	Potent cytotoxic effect in a micromolar range against T98, U87 and RT2, MCF7, EAC, and UACC cancer cell lines	[193]
	TSV-DM	*Trimeresurus stejnegeri *	Apoptosis, inhibitor of cell proliferation and inducer cell morphologic changes.	[194]

Disintegrin	Contortrostatin	*Agkistrodon contortrix contortrix *	Anti-invasive and antiadhesive activities on tumor cells and endothelial cells. Antitumor, antiangiogenic activities and inhibitor of tumor growth.	[171–175]
	Leucurogin	*Bothrops leucurus *	Anti-angiogenesis effect	[195]
	Saxatilin	*Gloydius saxatilis *	Inhibitor of tumor growth.	[181]
	Obtustatin	*Vipera lebetina *	Anti-angiogenesis effect	[165]
	Adinbitor	*Agkistrodon halys stejneger *	Inhibits angiogenesis (*in vitro *and *in vivo*)	[196]
	Albolabrin	*Trimeresurus albolabris *	Inhibits RGD-dependent integrins and metastasis	[169]
	Rhodocetin	*Calloselasma rhodostoma *	Inhibits the cell adhesion, migration, and collagen contraction	[197]
	Salmosin	*Agkistrodon halys brevicaudus *	Antiangiogenic and antitumorigenic	[88, 179, 180, 185]
	Trigramin	*Trimeresurus gramineus *	Inhibits the adhesion melanoma cells to fibronectin and fibrinogen.	[164, 166]
	Triflavin	*Trimeresurus flavoviridis *	Inhibits adhesion and migration cell and angiogenesis.	[168]
	Rhodostomin	*Agkistrodon rhodostoma *	Inhibits angiogenesis and grow tumor cell adhesion.	[163, 164, 177]
	Echistatin	*Echis carinatus *	Inhibits the adhesion of melanoma cells to extracellular matrix components	[170]

Serineproteases	Crotalase	*Crotalus adamanteus *	Inhibition of tumor growth	[153]
	Batroxobin	*B. moojeni *	Antimetastatic effect	[183]

Lectins	BjcuL	*B. jararacussu *	Cytotoxic effects and inhibits cell adhesion	[117]
	Lebectin and Lebecetin	*Macrovipera lebetina *	Inhibits adhesion, migration, and invasion of tumor cells; inhibits angiogenesis	[119]
	EM16	*Echis multisquamatus *	Cytoskeleton disassembly; inhibits adhesion and migration of HUVEC cells	[198]

Peptides	Cardiotoxin III (CTX III)	*Naja naja atra *	Blocks migration and invasion of MDA-MB-231 breast cancer cells	[42, 108]
	Cytotoxin P4	*Naja n. nigricollis *	Cytotoxicity	[40, 47, 48]
	Cathelicidin-BF	*Bungarus fasciatus *	Inhibits B16F10 and B16 proliferation	[199]

Inhibitors	BJ46a	*B. jararaca *	Inhibits the invasion and metastasis of tumor cells B16F10, a melanoma cell line, and MHCC97H, a human hepatocellular carcinoma cell line	[200]
	PIVL	*Macrovipera lebetina transmediterranea *	Serine protease inhibitor; exhibits an anti-tumor effect and displays integrin inhibitory activity without being cytotoxic. Inhibit the adhesion, migration, and invasion of human glioblastoma U87 cells.	[201]

Crude		*Montivipera xanthina *	Cytotoxic effect	[202]
	WEV—venom extracted	*Walterinnesia aegyptia *	Induction of apoptosis	[203]
		*Vipera lebetina turanica *	Inhibits the growth of human ovarian cancer through induction of apoptosis	[204]

## Abbreviations

svMP: Snake venom metalloproteases
svSP: Snake venom serineproteases
svPLA_2_s: Snake venom phospholipases A_2_
svLAAO: Snake venom L-amino acid oxidases
svCTL: Snake venom C-type lectin-like
CVF: Cobra venom factor.
